# Early discrimination of cognitive motor dissociation from disorders of consciousness: pitfalls and clues

**DOI:** 10.1007/s00415-020-10125-w

**Published:** 2020-08-04

**Authors:** Alessandro Pincherle, Frederic Rossi, Jane Jöhr, Vincent Dunet, Philippe Ryvlin, Mauro Oddo, Nicolas Schiff, Karin Diserens

**Affiliations:** 1 Acute Neuro-rehabilitation Unit, Department of Clinical Neurosciences, Lausanne University Hospital and University of Lausanne, Bâtiment Champ de l’Air, Rue du Bugnon 21, 1011 Lausanne, Switzerland; 2 Neurology Unit, Department of Medicine, Hopitaux Robert Schuman, Luxembourg, Luxembourg; 3 Department of Diagnostic and Interventional Radiology, Lausanne University Hospital and University of Lausanne, Lausanne, Switzerland; 4 Intensive Care Unit, Lausanne University Hospital and University of Lausanne, Lausanne, Switzerland; 5 Feil Family Brain and Mind Research Institute, Weill Cornell Medicine, New York, NY USA

**Keywords:** Disorders of consciousness, Cognitive motor dissociation, Brain injury, Motor behavior tool, Coma Recovery Scale

## Abstract

Bedside assessment of consciousness and awareness after a severe brain injury might be hampered by confounding clinical factors (i.e., pitfalls) interfering with the production of behavioral or motor responses to external stimuli. Despite the use of validated clinical scales, a high misdiagnosis rate is indeed observed. We retrospectively analyzed a cohort of 49 patients with severe brain injury admitted to an acute neuro-rehabilitation program. Patients’ behavior was assessed using the Motor Behavior Tool and Coma Recovery Scale Revised. All patients underwent systematic assessment for pitfalls including polyneuropathy and/or myopathy and/or myelopathy, major cranial nerve palsies, non-convulsive status epilepticus, aphasia (expressive or comprehensive), cortical blindness, thalamic involvement and frontal akinetic syndrome. A high prevalence (75%) of pitfalls potentially interfering with sensory afference (polyneuropathy, myopathy, myelopathy, and sensory aphasia), motor efference (polyneuropathy, myopathy, motor aphasia, and frontal akinetic syndrome), and intrinsic brain activity (thalamic involvement and epilepsy) was found. Nonetheless, the motor behavior tool identified residual cognition (i.e. a cognitive motor dissociation condition) regardless of the presence of these pitfalls in 70% of the patients diagnosed as unresponsive using the Coma Recovery Scale Revised. On one hand, pitfalls might contribute to misdiagnosis. On the other, it could be argued that they are clues for diagnosing cognitive motor dissociation rather than true disorders of consciousness given their prominent effect on the sensory–motor input–output balance.

## Introduction

One of the main challenges in neurology, often implying serious ethical consequences, is the reliable bedside clinical identification of consciousness in patients with severe brain injury [1, 2]. Functional neuroimaging can help detect residual cognitive function and awareness in some patients who appear entirely unresponsive at the bedside [3], pointing to the so-called cognitive motor dissociation (CMD) condition. This approach has, however, a limited feasibility in the neuro-intensive-care setting. Consequently, assessment of motor/verbal/visual responses to external stimuli remains the current standard in clinical practice [4], with the Coma Recovery Scale Revised (CRS-R) regarded as the gold-standard for diagnosis of disorders of consciousness (DOC) [5]. However, despite the use of validated clinical scales, an unacceptably high rate of clinical misdiagnosis is observed [6].

We recently developed a simple bedside clinical tool, the motor behavior tool (MBT) revised to its current form (MBT-r), to refine the evaluation of residual cognition in patients at an early stage after severe brain injury [7, 8]. We found that the MBT detected signs of residual cognition in almost two-thirds of patients who had been classified as unconscious using the CRS-R, suggesting that the MBT can identify a subgroup of patients with blocked motor efference/output rather than a true consciousness disorder. The high percentage of these recognized patients suggests the presence of common confounding factors that mask their accurate diagnosis, similar to the many specific factors that hamper accurate assessment of brain death exams [9]. Indeed, several factors including co-morbidities/complications that interfere with the production of appropriate behavioral or motor responses to external stimuli might affect clinical examination in the neuro-critical care setting. Moreover, patients with severe brain injury suffer from neurological deficits that often render any movement slight and inconsistent and patients become easily fatigued. These obstacles to behavioral evaluation might lead to the erroneous diagnosis of DOC.

Here, we aimed to describe the confounding clinical factors that might interfere with the clinical recognition of consciousness, and thus increase the uncertainty of clinical evaluation. Specifically, we sought to: (a) identify and describe confounding clinical factors (here defined as pitfalls) that interfere with clinical/behavioral evaluation, (b) assess the effects of these pitfalls on the diagnostic classification according to CRS-R and MBT scales, and (c) suggest simple clinical and paraclinical clues to help overcome pitfalls, which hinder recognition of residual cognition.

## Methods

### Patients

The local ethical committee approved this retrospective cohort study (Ref. 142-09). We consecutively enrolled 49 patients (26 men, aged 51.8 ± 19.5 years) who were admitted to the Lausanne University Hospital Acute Neuro-rehabilitation Unit between June 2012 and December 2017 after severe brain injury (traumatic and non-traumatic injuries, including vascular, anoxic, encephalopathic, and neoplastic disorders) requiring the initial treatment in the neuro-intensive-care unit (Table 1). We excluded patients with the following conditions at the time of behavioral assessments: hypo- and hyper-glycemia (70 and 200 mg/dL, respectively); hypo- and hyper-natremia (133 and 150 mmol/L, respectively); renal or liver failure.

**Table 1:** Patients demographics and clinical characteristics

ID	Sex	Age	Etiology	Time to clinical appraisal (days)	Time to rehab (days)	Rehab stay (days)	CRS-R diagnosis	MBT diagnosis	Negative MBT signs	Pitfalls
1	F	17	Traumatic	20	33	34	Coma/UWS	True DOC	Yes	Aphasia
2	M	17	Traumatic	15	18	14	MCS/EMCS	CMD	No	NCSE
3	M	37	Anoxic	8	17	26	Coma/UWS	True DOC	Yes	None
4	F	74	Traumatic	5	17	17	Coma/UWS	CMD	No	Frontal Akinetic Syndrome
5	F	78	Vascular	6	26	21	MCS/EMCS	CMD	No	Frontal Akinetic Syndrome
6	F	67	Traumatic	13	19	15	Coma/UWS	CMD	No	None
7	F	40	Vascular	3	26	33	Coma/UWS	True DOC	No	Thalamic Involvement
8	M	78	Infectious	19	48	43	Coma/UWS	CMD	No	NCSE
9	M	59	Vascular	8	29	29	Coma/UWS	CMD	No	Neuropathy–Myopathy
10	M	18	Traumatic	15	28	41	Coma/UWS	True DOC	Yes	None
11	F	54	Vascular	14	23	41	Coma/UWS	True DOC	Yes	None
12	M	45	Traumatic	11	31	9	Coma/UWS	CMD	No	Neuropathy–Myopathy
13	F	67	Vascular	18	26	22	Coma/UWS	CMD	No	Frontal Akinetic Syndrome
14	M	24	Traumatic	27	38	30	Coma/UWS	CMD	No	None
15	F	58	Traumatic	13	38	20	MCS/EMCS	CMD	No	NCSE
16	F	54	Traumatic	28	21	34	Coma/UWS	CMD	Yes	Aphasia
17	M	24	Vascular	9	18	35	Coma/UWS	True DOC	Yes	None
18	M	27	Traumatic	14	18	14	Coma/UWS	CMD	No	Frontal Akinetic Syndrome
19	F	65	Anoxic	9	31	20	Coma/UWS	CMD	No	Neuropathy–Myopathy
20	M	21	Traumatic	5	12	21	Coma/UWS	CMD	No	None
21	F	23	Traumatic	20	33	8	Coma/UWS	True DOC	Yes	Aphasia
22	M	50	Anoxic	15	22	50	MCS/EMCS	CMD	No	Neuropathy–Myopathy
23	M	43	Traumatic	10	19	35	Coma/UWS	CMD	No	None
24	F	84	Traumatic	3	21	19	Coma/UWS	CMD	No	Neuropathy–Myopathy
25	M	80	Traumatic	2	10	32	Coma/UWS	CMD	No	Frontal Akinetic Syndrome
26	M	43	Vascular	18	34	20	Coma/UWS	CMD	No	Aphasia
27	M	73	Traumatic	23	28	29	Coma/UWS	CMD	No	Aphasia
28	F	37	Traumatic	8	49	41	Coma/UWS	True DOC	Yes	Thalamic Involvement
29	M	34	Traumatic	8	15	33	Coma/UWS	CMD	No	Aphasia
30	F	61	Vascular	12	19	56	Coma/UWS	CMD	No	Aphasia
31	M	61	Vascular	12	26	41	Coma/UWS	CMD	No	Aphasia
32	F	69	Toxic-Metabolic	24	36	64	MCS/EMCS	CMD	No	Neuropathy–Myopathy
33	F	62	Vascular	6	51	33	MCS/EMCS	CMD	No	Aphasia
34	F	66	Traumatic	4	19	39	Coma/UWS	CMD	No	Frontal Akinetic Syndrome
35	M	35	Toxic-Metabolic	17	17	27	Coma/UWS	True DOC	No	None
36	M	42	Traumatic	16	23	22	Coma/UWS	CMD	No	NCSE
37	F	75	Vascular	11	25	9	MCS/EMCS	CMD	No	Aphasia
38	M	61	Vascular	3	15	47	Coma/UWS	CMD	No	Frontal Akinetic Syndrome
39	M	73	Traumatic	11	25	59	Coma/UWS	CMD	No	Frontal Akinetic Syndrome
40	F	35	Traumatic	18	33	33	Coma/UWS	True	Yes	None
41	F	42	Vascular	5	13	34	Coma/UWS	CMD	No	Aphasia
42	M	55	Vascular	1	24	36	MCS/EMCS	CMD	No	Frontal Akinetic Syndrome
43	M	59	Anoxic	20	26	20	Coma/UWS	True DOC	Yes	None
44	F	24	Traumatic	6	15	24	MCS/EMCS	CMD	No	Frontal Akinetic Syndrome
45	M	71	Anoxic	12	61	41	MCS/EMCS	CMD	No	None
46	F	52	Vascular	6	19	31	MCS/EMCS	CMD	No	Aphasia
47	F	72	Vascular	2	23	28	Coma/UWS	CMD	No	Frontal Akinetic Syndrome
48	M	73	Vascular	7	14	43	Coma/UWS	CMD	No	Frontal Akinetic Syndrome
49	M	63	Vascular	18	34	48	MCS/EMCS	CMD	No	Frontal Akinetic Syndrome

*CRS-R* Coma Recovery Scale Revised, *MBT* motor behavior tool, *MCS* minimally conscious state, *UWS* unresponsive wakefulness syndrome, *EMCS* emergence from minimally conscious state, *CMD* cognitive motor dissociation, *DOC* disorder of consciousness, *NCSE* non-convulsive status epilepticus

### ***Clinicalandimagingassessment***

Patients underwent neurobehavioral assessment as early as 48 h after therapeutic sedation withdrawal and within 30 days of the brain injury using the French version of the CRS-R based on the existing guidelines [10, 11] complemented by the MBT [7, 8]. As previously described in detail in [8], the MBT brings out clinical signs of preserved conscious awareness by means of careful evaluation and scoring of subtle motor behavior not adequately identified using the CRS-R only, due to severe motor defects. The MBT uses a simple dichotomous scoring method, validated in a blinded study [8], to identify “positive” signs of residual cognition/conscious awareness and “negative” signs of possible brain-stem dysfunction (Table 2). Patients showing signs of residual cognition at MBT are classified as having “clinical CMD” condition.

**Table 2:** The motor behavior tool revised

	Item	Notes/instructions
*Positive* *signs*		
1	Spontaneous non-reflexive movements	Observation of the patient without any stimulation. At least one non-reflexive movements defined as intentional motor pattern non-stereotypical, not contextualized and non-repetitive
2	Response to command	Any scorable response to verbal command
3	Visual fixation or visual pursuit	Any clearly discernible visual fixation or visual pursuit in any direction
4	Responses in a motivational context	Any increased in the frequency of non-reflexive motor responses in a salient context (e.g., mother tongue, patient's own name)
5	Defensive non-reflexive response to a noxious stimulation—Nipple	Twisting the patient's nipple while keeping the patient's healthier arm between the patient's body and the examiner's arm. Any attempt to push away the examiner's arm that is not a stereotypical posture involving extension and internal rotation of the arms
6	Defensive non-reflexive response to a noxious stimulation—Nail bed	Deep pressure to nail beds of four extremities. Any limb movement whose kinematics differs from a motor reflex response in terms of orientation planes and the type of elicited muscles is scored as defensive.
7	Response to a noxious stimulation—Grimace	Observation of at least one grimace during administration of noxious stimulation
*Negative* *signs*		
8	Abnormal motor or neurovegetative responses to stimulation	Observation of slow, stereotyped flexion or extension of the upper and/or lower extremities after noxious stimulation or neurovegetative responses (i.e., tachycardia, hypo/hyper-ventilation, hypertension, excessive sweating) to stimulation.
9	Signs of roving eyes or absence of oculocephalic reflex	Slowly roving eyes movements are typical of metabolic encephalopathy indicating diffuse cerebral dysfunction. Oculocephalic responses imply intact brain-stem pathways

Patients were grouped as MCS/EMCS (minimally conscious state/emerging from the minimally conscious state) or coma/UWS (unresponsive wakefulness syndrome) based on the CRS-R and as potential clinical CMD (i.e., presenting signs of residual cognition) or true-DOC (i.e., presenting no signs of residual cognition) based on the MBT.

Patients underwent a morphological brain magnetic resonance imaging (MRI) during their hospitalization in the Intensive Care or Acute Neuro-rehabilitation Unit (time from admission to the Acute Neuro-rehabilitation Unit: median 8.0 days, range min–max: 0–42 days). MRI acquisitions included T1-weighted, T2-weighted or fluid attenuated inversion recovery, T2 gradient echo or susceptibility-weighted, and diffusion-weighted and T1-weighted images after intravenous injection of gadolinated contrast media.

A neuroradiologist (VD) blinded to the results of MBT/CRS-R assessments, performed a detailed evaluation of all morphological brain MR images. Lesions in frontal, temporal, parietal, occipital, basal ganglia, thalamus, hypothalamus, mesencephalon, pons, and cerebellum regions were identified bilaterally and recorded. Special attention was given to score lesions in areas strategic for vision (calcarine cortex [12, 13]), language (dominant fronto-temporo-parietal cortices involved in language [14, 15]), arousal control (thalamic involvement [16, 17]), motor planification and execution (frontal–subcortical circuitry [18]), and cranial nerve nuclei in the brain stem [19, 20].

### ***Confoundingclinicalfactorsorpitfalls***

We identified neurological deficits/co-morbidities (i.e. pitfalls), intrinsically related to the brain injury process and, which might potentially interfere with the ability to deliver motor and verbal responses to external stimulation during the neuro-behavioral assessment. The choice of these co-morbidities was reached from a review of the literature and discussion in focus groups with experts in intensive-care and severe brain injury management. The following seven conditions were identified as pitfalls: polyneuropathy and/or myopathy and/or myelopathy, major cranial nerve palsies, non-convulsive status epilepticus (NCSE), aphasia (expressive or comprehensive), cortical blindness, thalamic involvement, and frontal akinetic syndrome (Table 3 presents the extensive clinical/paraclinical criteria used to identify the pitfalls).

**Table 3:** Pitfalls description

Comorbid condition/pitfall (references)	Signs/clues to identify pitfalls	
	Clinical	Para-clinical
Polyneuropathy or myopathy	Areflexia, amyotrophia, flaccidity	ENG/EMG—nerve conduction and electromyographic abnormalities
Cranial nerves palsies [19, 20]	Cranial nerve palsies	Imaging (MRI)—brain-stem nuclear or nerve lesions
Non convulsive status epilepticus	Staring, eye deviations, neglect, myoclonus	EEG—epileptic potentials
Cortical blindness [12, 13]	Absence of visual interaction, absence of menace reflex, absence of visual pursuit	Imaging (MRI)—bilateral occipital lesions
Akinetic (frontal) Syndrome [18]	Marked reduction of spontaneous movement and speech production with inconstant visual fixation and tracking, inconstant command-following and vocalization	Imaging (CT or MRI)—(Bi-) cortico-subcortical frontal, cerebellar, basal ganglia lesions
Thalamic involvement [16, 17]	Intermittent vigilance fluctuations, short-lasting (i.e., seconds–minutes) easily reverted with sensory stimulation	Imaging (MRI)—thalamic lesions or hypothalamic lesions
Aphasia [14, 15]	Anarthric or mutic patient, absence of command-following	Imaging (MRI or CT)—dominant sided parietal-fronto-temporal lesions

After the neuro-radiological assessment and according to the diagnostic criteria listed in Table 3, a neurologist (FR) not involved in MBT/CRS-R administration systematically reviewed all patient records to identify the presence of any of the seven pitfalls present at the time of MBT/CRS-R testing.

### Statistical analysis

Statistical analyses were performed using the Jasp software. Continuous variables are presented as mean ± standard deviation or median (range min–max). Categorical variables are presented as number and percentage. We compared categorical variables using 2 × 2 contingency tables and the Chi-squared test with Fisher’s correction applied when necessary. We used the Student’s *t* test to compare the means of continuous/ordinal variables after verifying normal distribution.

Results were considered significant at *p* < 0.05.

## Results

### Residual cognition and diagnosis

Among the 49 enrolled patients, 23 had a traumatic, 18 a vascular, 5 an anoxic, 2 a toxic–metabolic, and 1 an infectious etiology. The mean time to CRS-R/MBT assessment after injury was 11 days (range 1–28), while mean time from admission to the acute rehabilitation unit was 25 days (range 10–61). The mean stay in the unit was 31 days (range 8–64).

Based on the CRS-R, 37 patients were diagnosed as coma/UWS and 12 MCS/EMCS. In contrast, using the MBT, 11 patients were diagnosed as true DOC (i.e., without signs of residual cognition) and 38 as clinical CMD (i.e., with signs of residual cognition).

Of the 37 patients classified as coma/UWS using the CRS-R, 26 (70%) showed signs of residual cognition based on the MBT. Furthermore, all 12 (100%) patients classified as MCS/EMCS regarding the CRS-R showed signs of residual cognition performing the MBT.

Negative MBT test signs (signs of possible brain-stem dysfunction) were identified in 10 patients (20%); only one of these patients was diagnosed as clinical CMD.

### Confounding clinical factors/pitfalls

We observed pitfalls likely able to interfere with identification of behavioral signs of cognition in 37 patients (75%). Specifically, as pitfalls, we detected 13 akinetic frontal syndrome patients (26%), 12 aphasia (24%), 6 neuropathy/myopathy (12%), 4 NCSE (8%), and 2 patients with thalamic involvement (4%). None of the patients presented with cortical blindness or major cranial nerve dysfunction. A total of 15 patients (30%) had more than one pitfall.

### Pitfalls and diagnostic classification

**Fig. 1:**
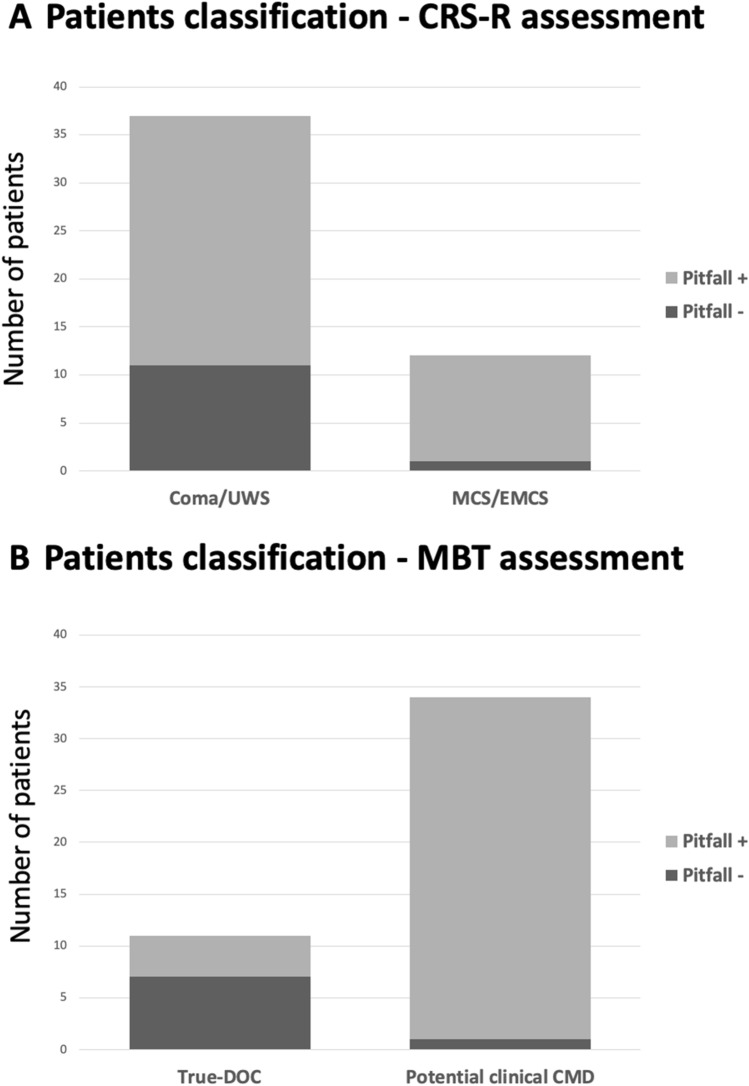
**a** CRS-R classification and pitfall identification. On the left, the bar shows the number of Coma/UWS patients presenting with (light gray) or without (dark gray) pitfalls; the right bar shows the number of MCS/EMCS patients presenting with (light gray) and without (dark gray) pitfalls. **b** MBT classification and pitfall identification. On the left, the bar shows the number of true-DOC patients presenting with (light gray) and without (dark gray) pitfalls; the right bar shows the number of potential clinical CMD patients presenting with (light gray) and without (dark gray) pitfalls. *UWS* unresponsive wakefulness syndrome, *MCS* minimally conscious state, *EMCS* emergence from minimally conscious state, *DOC* disorder of consciousness, *CMD* cognitive motor dissociation

From the CRS-R classification, we noticed pitfalls in 11/12 (91%) of the patients with MCS/EMCS and 26/37 (70%) of the patients with coma/UWS (*p* = 0.13) (Fig. 1a).

On the contrary, with the MBT classification, pitfalls were recognized in only 4/11 patients (36%) with true-DOC and 33/38 patients with potential clinical CMD (86%) (*p* < 0.001) (Fig. 1b).

## Discussion

In this retrospective cohort study on patients with severe brain injuries admitted to an acute neuro-rehabilitation program, we found a high prevalence (75%) of patients with clinical conditions potentially interfering with a behavioral response to sensory stimulation. Despite the high predominance of these so-called pitfalls, we found that signs of residual cognition could be identified using the MBT in 70% of patients diagnosed as coma or UWS using the CRS-R. This indicates the ability of the MBT to identify subtle motor behavior and that MBT test sensitivity is unaffected by the high presence of pitfalls that interfere with sensory afference (polyneuropathy, myopathy, myelopathy, and sensory aphasia), motor efference (polyneuropathy, myopathy, motor aphasia, and frontal akinetic syndrome), or intrinsic brain activity (thalamic involvement and epilepsy).

Unlike the MBT, the CRS-R appeared to be affected by pitfalls when used at a very early phase. Specifically, the CRS-R, mainly based on residual motor output, might be insufficient to assess the cognitive ability to interact in patients with impaired motor efference/output, lesions affecting strategic functional areas of the central nervous system, or altered function of the peripheral nervous system. In line with this, it was suggested that performing at least five CRS-R assessments in patients with DOC within a 2-week time interval might reduce the misdiagnosis rate [21]. However, while performing repeated tests might be feasible in stabilized patients; it might not be suitable in the intensive-care setting where rapid decisions are required.

In this study, we found three pitfalls, frontal akinetic syndrome, aphasia, and polyneuropathy, which could, in theory, coexist in a single patient, present in 60% of our patient population. Therefore, they deserve further specific consideration.

Frontal akinetic syndrome, which shares some characteristics of akinetic mutism [22–24], is a condition in which patients might appear outwardly attentive and vigilant (often shown by deliberate visual tracking), but exhibit a paucity of behavioral responses, even when prompted by external stimuli. The brain injury pattern most commonly associated with this syndrome is bilateral damage of the anterior medial regions of the frontal cortex. Notably, all the patients (13/13) in our cohort with this pitfall recovered consciousness at discharge and presented signs of cognition in the early MBT evaluation (mean days after injury, 7; range 1–18). Indeed, the MBT seemed to have sufficient sensitivity for subtle motor phenomena (such as grimaces, spontaneous movements, or defensive responses to pain) that might persist in patients with CMD. On the contrary, 9 out of the 13 patients with frontal akinetic syndrome were classified as unconscious (coma/UWS) using the CRS-R. Therefore, detailed imaging analysis aiming to identify frontal cortico-subcortical motor pathway disconnections [18] might be crucial to minimize residual cognition underestimation at the very early stage after brain injury.

We found that 18% of our patients had aphasia, which is consistent with the findings of a previous series [25]. The existence of overlooked language disorders might negatively interact with a behavioral response to verbal commands, leading to underestimation of the patient’s level of consciousness, especially when using scales like CRS-R that require persistent responses. The possibility of co-occurrence of aphasia and a disorder of consciousness should particularly be considered in the context of left hemisphere focal (cerebrovascular or post-traumatic) lesions in the dominant peri-sylvian area [14, 15]. The presence of focal brain lesions here, their extent and localization, can be easily confirmed by systematic structural brain imaging examination using computer tomography or MRI scans. Functional neuroimaging appears to be particularly promising for disentangling impaired consciousness and aphasia [25].

Previous studies reported that 40–80% of critically ill patients present with acute poly-neuro-myopathy [26, 27]. Under these circumstances, it might be difficult to clinically identify the absence of motor behavioral signs due to central causes. Therefore, it is important to carefully evaluate osteo-tendon reflexes and muscular tonus with complementary electroneuromyography to assess peripheral nerve and muscle function.

Earlier studies from our group suggested that the MBT, which detects subtle motor behaviors missed by the CRS-R, can clinically discriminate patients with potential clinical CMD from those with true DOC [7, 8, 28]. The MBT was reported to detect residual cognition at very early stages after brain injury [7, 8] and the present study showed consistent findings despite the presence of pitfalls. Our data, based on careful clinical observation, are in line with increasing evidence of residual brain activation in unresponsive patients detected using more complex electroencephalographic (EEG) or functional neuroimaging protocols [29–31]. Indeed, although the original definition of CMD is based on this clinical/paraclinical discordance [3], there is still no gold-standard definition for CMD. Moreover, successful completion of complex behavioral paradigms requires considerable preservation of working memory, attention, and executive functions, such that the clinical spectrum of CMD is likely to be wide, including many patients who cannot perform these tasks reliably yet show behavioral evidence of awareness, akin to the patients identified in the present study. In this respect, considering pitfalls potentially able to interfere with command-following approaches is extremely relevant and might influence positively the detection rates of CMD by subsequent EEG or functional MRI protocols. Indeed, approaches based on direct measurements of cortico-cortical connectivity using transcranial magnetic stimulation (TMS)-EEG have been proven to discriminate MCS from UWS [32, 33]. Furthermore, they are theoretically unaffected by pitfalls; however, TMS-EEG has only been assessed in chronic patients and its ability to detect covert residual cognition at an early phase and feasibility in the ICU setting remain unknown.

**Fig. 2:**
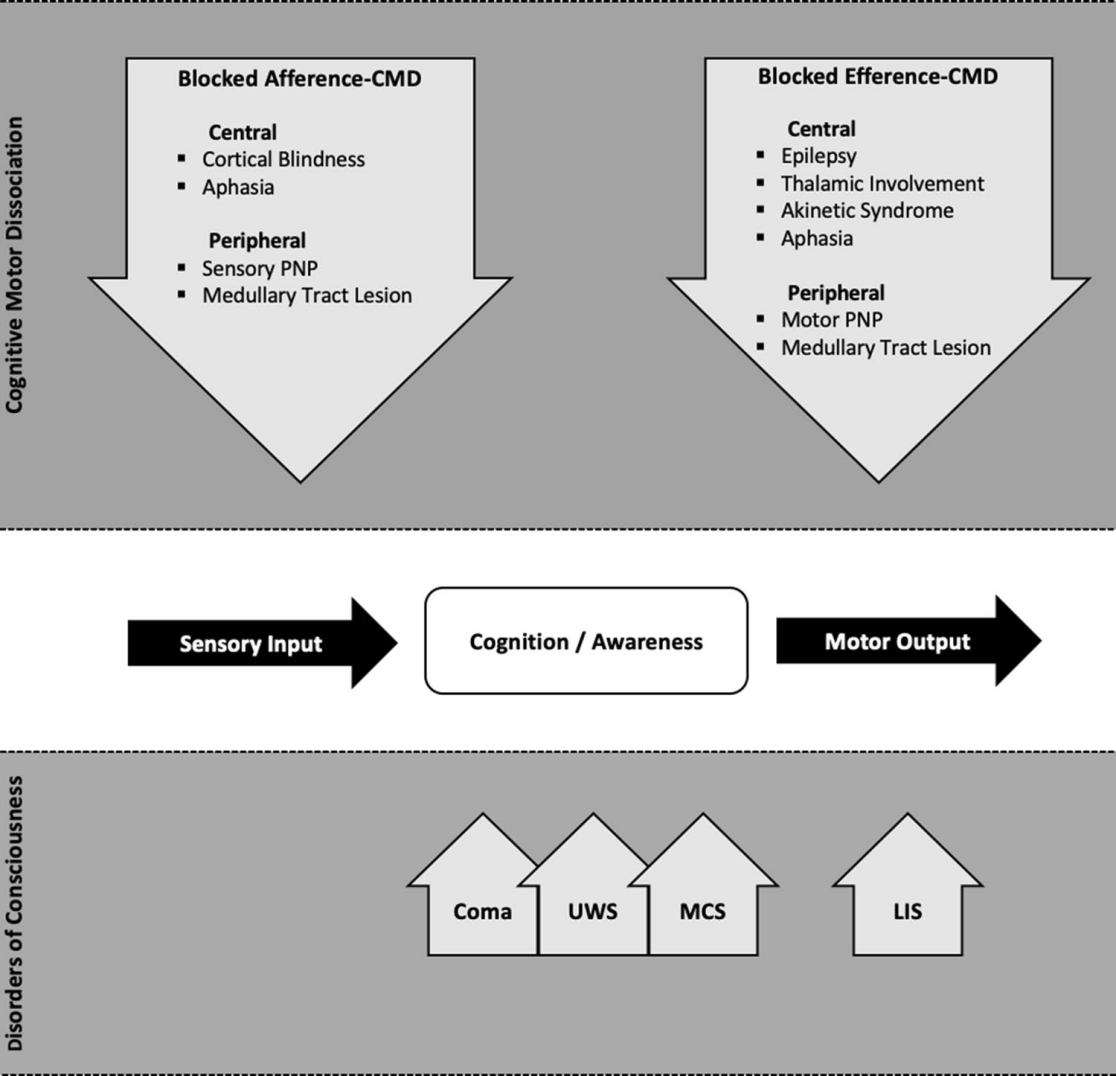
Pitfall interference on sensory-motor output and intrinsic brain activity. Several arguments point to distinguishing CMD as a separate entity from disorders of consciousness, the latter being more the expression of widespread cortico-thalamic dysregulation directly impairing cognition and awareness. LIS is characterized by preserved consciousness and cortical functions and a complete motor output disruption. CMD reflects rather a dysfunction of the motor planning cortico-subcortical circuitries, with pitfalls negatively interacting with sensory input and/or motor output. *CMD* cognitive motor dissociation, *PNP* polyneuropathy, *UWS* unresponsive wakefulness syndrome, *MCS* minimally conscious state, *LIS* locked-in syndrome, *DOC* disorders of consciousness

There are several theoretical arguments describing CMD and DOC as two separate entities. According to the meso-circuit hypothesis, CMD patients probably have some functional disturbance of the forebrain systems (frontal/prefrontal, cortical-striato-pallidal, and thalamocortical loop systems) associated with motor preparation and action [34], unlike the situation in locked-in subjects, producing both fluctuations in responsiveness and varying limitations of motor control. This hypothesis suggests, therefore, that pitfalls have a relevant and direct interference on sensory–motor output and intrinsic brain activity; indeed, they could be considered CMD features rather than confounding factors (Fig. 2). Contrastingly, only widespread cortico-thalamic damage/disconnection can produce true DOC [35, 36] based on the view that conscious states do not rely on a single cortical area or network, but rather require sustained, complex, and differentiated brain-scale communication defects [37–39]. Consistent with this hypothesis, we found a higher prevalence of cortical laminar necrosis and diffuse axonal injuries [40, 41] among patients with DOC compared to patients with clinical CMD (data not shown). However, as we did not design this study to address imaging patterns, this finding should be regarded cautiously and requires further analysis. In addition, it should be recognized that hypoxic–ischemic injuries and diffuse axonal injury harbor subtypes more likely to end up as CMD although infrequent in demographic distribution. Examples include diffuse axonal injury with predominant radial component disrupting brain-stem axons [42] and hypoxic injuries producing dysfunction of the motor cortex or basal ganglia with preservation of global integrative brain function [43].

Another finding that supports the discrimination of CMD from DOC is also the observed between-group difference in the frequency of negative signs measured with the MBT (90% vs. 10%). Negative signs observed in MBT testing (roving eyes or absence of oculocephalic reflex and abnormal motor or neurovegetative responses to stimulation) were interpreted as indicators of major brain-stem involvement. The higher prevalence of negative signs from the MBT among patients with DOC is expected given the role of the brain-stem arousal system as the physiological foundation of forebrain arousal control, the base of classical and modern DOC patho-physiology [44, 45].

The present study has several limitations. First, although supported by repeated observations [7, 8, 28], we categorized patients as presenting with potential clinical CMD only on clinical observations from MBT rating. We did not perform active mental-imagery tasks according to the acknowledged operational definition of CMD to confirm our clinical diagnosis [4]. We have, however, previously shown that multisensory processing of the peri-personal space (the multisensory-motor space immediately surrounding the body) was preserved in putative (based on MBT) CMD but not DOC patients [46]. Further studies are warranted to provide an objective measure of covert awareness/residual signs of cognition combining functional MRI or EEG testing and the MBT. Second, we did not validate the identification of pitfalls in an independent study. However, we based their identification on literature-based (see Table 3 for details) clinical/paraclinical criteria routinely used in our institution; furthermore, the longitudinal observation during the hospital stay allowed us to confirm clinically the presence of pitfalls putatively identified at the very early stage after brain injury using paraclinical tests. We, therefore, believe that lack of validation of our pitfall criteria did not affect our results. Third, the unbalanced sample in terms of etiology (high prevalence of traumatic and vascular compared to other etiologies) might limit the generalization of our findings to different populations. A preliminary analysis of our cohort did not show any difference (data not shown), but a larger sample is needed to stratify the results according to different etiological groups.

## Conclusions

We found a high prevalence of pitfalls that could potentially interfere with production of behavioral signs when performing clinical diagnosis of DOC. Furthermore, our findings indicate that the MBT can discriminate patients with clinical CMD from those with true DOC regardless of the presence of pitfalls. A systematic global clinical screening of pitfalls (including a critical structural imaging revision to reveal potential pitfalls) is needed in the quest to minimize misdiagnosis in cases of suspected DOC. On one hand, pitfalls could contribute to misdiagnosis. On the other hand, they may point towards a potential clinical CMD diagnosis given their prominent effect on the sensory–motor input–output balance.
